# Dynamic CT Myocardial Perfusion: The Role of Functional Evaluation in the Diagnosis of Coronary Artery Disease

**DOI:** 10.3390/jcm12227062

**Published:** 2023-11-13

**Authors:** Agata Zdanowicz, Maciej Guzinski, Michal Pula, Agnieszka Witkowska, Krzysztof Reczuch

**Affiliations:** 1 Department of General Radiology, Interventional Radiology and Neuroradiology, Wroclaw Medical University, Borowska 213, 50-556 Wroclaw, Poland; maciej.guzinski@umw.edu.pl; 2 Lower Silesian Oncology, Pulmonology and Hematology Center, Hirszfelda Square 12, 53-413 Wroclaw, Poland; 3 Institute of Heart Diseases, University Clinical Hospital in Wroclaw, Borowska 213, 50-556 Wroclaw, Polandkrzysztof.reczuch@umw.edu.pl (K.R.); 4 Department of Cardiology, Faculty of Medicine, Institute of Heart Diseases, Wroclaw Medical University, 50-367 Wroclaw, Poland

**Keywords:** dynamic myocardial perfusion computed tomography, coronary computed tomography angiography, coronary artery disease, coronary stenosis, myocardial ischemia

## Abstract

Coronary computed tomography angiography (CTA) is a widely accepted, non-invasive diagnostic modality for the evaluation of patients with suspected coronary artery disease (CAD). However, a limitation of CTA is its inability to provide information on the hemodynamic significance of the coronary lesion. The recently developed stress dynamic CT perfusion technique has emerged as a potential solution to this diagnostic challenge. Dynamic CT myocardial perfusion provides information on the hemodynamic consequences of coronary stenosis and is used to detect myocardial ischemia. The combination of stress dynamic CT myocardial perfusion with CTA provides a comprehensive assessment that integrates anatomical and functional information. CT myocardial perfusion has been validated in several clinical studies and has shown comparable accuracy to Positron Emission Tomography (PET) and stress magnetic resonance imaging (MRI) in the diagnosis of hemodynamically significant coronary stenosis and superior performance to Single Photon Emission Computed Tomography (SPECT). More importantly, CTP-derived myocardial perfusion has been shown to have a strong correlation with FFR, and the use of CTP results in a reduction of negative catheterizations. In the context of suspected stable coronary artery disease, the CT protocol with dynamic perfusion imaging combined with CTA eliminates the need for additional testing, making it a convenient “one-stop-shop” method and an effective gatekeeper to an invasive approach.

## 1. Introduction

Coronary computed tomography angiography (CCTA) has gained widespread recognition as a non-invasive diagnostic modality for the evaluation of suspected coronary artery disease (CAD).

Due to its exceptional sensitivity in ruling out CAD [1], CCTA has been classified as a first-line test (Class I) in the low to intermediate risk category, according to the current guidelines endorsed by the European Society of Cardiology (ESC) [2].

However, a notable limitation of CCTA stems from its inability to provide information on the hemodynamic significance of the coronary lesion. This is particularly important given that the degree of stenosis is not always a reliable predictor of CAD severity [3].

In fact, a considerable proportion of moderate and severe stenoses do not demonstrate hemodynamic significance, as evidenced by a fractional flow reserve (FFR) value of greater than 0.8. Moreover, the FAME study reported that approximately one-fifth of mild lesions were found to have an FFR value indicative of ischemia (FFR < 0.8) [4]. Therefore, the exclusive reliance on visual assessment may contribute to unnecessary referrals for invasive diagnostic procedures [5,6].

Indeed, approximately 60% of patients who undergo invasive coronary angiography have no obstructive CAD [5].

In light of these discrepancies, the diagnostic approach has shifted from anatomic assessment to evaluation of the physiological impact of a coronary lesion on myocardial perfusion. The extent of myocardial ischemia has emerged as a key factor in the decision-making process for revascularization and has been shown to be more important than the severity of stenosis for the identification of patients who may benefit from percutaneous coronary intervention (PCI) [7]. This notion is reflected in current guidelines that support the benefits of ischemia-guided revascularization [2,8].

The need for a comprehensive approach has sparked a growing interest in the exploration of computed tomography myocardial perfusion imaging (CT-MPI), particularly due to the practical advantage of its potential conjunction with CCTA.

Dynamic CT-MPI can detect the presence of ischemia and provide information on the hemodynamic consequences of obstructive coronary disease [9,10]. Therefore, it holds promise for effective risk stratification of patients, potentially reducing the number of unnecessary invasive procedures.

Additionally, it offers incremental diagnostic value in challenging clinical scenarios, such as severe calcifications, where CCTA alone may not be sufficient for an accurate and precise diagnosis [11,12].

This review explores the principles, technical aspects, clinical significance, and the current state of the art of dynamic CT myocardial perfusion imaging.

## 2. General Overview of Myocardial CT Perfusion

Myocardial CTP serves as a tool for the assessment of hemodynamic changes in cardiac segments resulting from coronary lumen stenosis [9].

Evaluation of myocardial perfusion is premised on the distribution of the iodinated contrast through the myocardial tissue. Given the linear correlation between the CT attenuation value and the iodine concentration, coupled with the reliance on arterial blood circulation, a reduction in contrast concentration manifests as a hypoattenuating region indicative of a myocardial perfusion defect [9,13].

CTP can be acquired in static or dynamic mode. Static CTP is performed analogously to CCTA. A data set of images is obtained at a single time point. The assessment of defects remains qualitative in nature; regions of hypoattenuation (indicating hypoperfusion) are compared to normally perfused myocardial regions facilitating the identification of regions exhibiting compromised blood flow and perfusion abnormalities. Variations in attenuation values provide insights into the distribution and dynamics of blood supply to different areas of the myocardium [10,14].

On the other hand, dynamic myocardial perfusion involves acquiring a data set of images taken at multiple time points following the injection of iodine contrast. These temporal samplings of myocardial iodine distribution are utilized to establish time-attenuation curves (TACs) that are subsequently mathematically processed to compute perfusion parameters that provide estimates of absolute myocardial blood flow.

Dynamic CTP enables qualitative, semi-quantitative, and fully quantitative assessment of myocardial blood flow (MBF). The quantitative analysis is particularly advantageous as it can detect and reveal balanced ischemia in the setting of global reduction in blood flow, which might not be readily apparent using qualitative visual assessments alone. However, importantly, this imaging is associated with substantially higher radiation exposure [10,15].

Stress myocardial CT perfusion involves the use of a vasodilator stressor to induce hyperemia, allowing the assessment of hemodynamic responses in tissues under physiological stress [10,15].

## 3. Physiological Principles of Coronary Circulation

At rest, myocardial oxygen extraction is high, specifically in the range of 75 to 80% [16]. Therefore, as oxygen demand rises, the potential for further increase in the rate of oxygen extraction is limited and must be compensated for by an augmentation of coronary blood flow [16]. Autoregulation in healthy coronary circulation allows proportional dilation of the arterioles in response to elevated metabolic demand. This mechanism preserves a relatively stable coronary blood flow over a wide range of perfusion pressures [17].

In the presence of a stenosis, an increase in vascular resistance and a decrease in blood pressure distal to the stenotic site are observed. This triggers vasodilation, which reduces resistance and thus preserves resting blood flow until the stenosis reaches a critical size (85–90%). However, under maximal hyperemia, a stenosis of >45% initiates a decrease in coronary blood flow [17]. As atherosclerosis progresses and coronary reserve diminishes, the mismatch between myocardial oxygen supply and demand, known as the ischaemic cascade, is initiated.

Perfusion abnormalities are among the earliest manifestations of an evolving ischemic event, preceding wall motion abnormalities, ECG changes, and clinical symptoms [17].

## 4. Stressor Agent

There is a wide range of stressor agents that might be employed in stress dynamic CT-MPI, namely adenosine, dipyridamole, dobutamine, or regadenoson. Currently, adenosine and regadenoson are the two most frequently used vasodilators. Adenosine is administered as a continuous infusion, at a rate of 140 mcg/kg/min, over a period of 3 to 5 min; conversely, regadenoson is given as a single bolus at a fixed dose of 400 mg over 10 s [18].

Adenosine non-selectively activates four subtypes of A receptors: A1, A2A, A2B, and A3. Stimulation of A2 receptors induces coronary and systematic vasodilatation, whereas binding to A1 receptors mediates the suppression of the sinus (SA) and atrioventricular (AV) nodes. Furthermore, activation of A3 results in airway smooth muscle constriction [18,19,20,21]. Consequently, the application of adenosine is linked to a broad range of undesired side effects, including AV block, bronchospasm, and peripheral vasodilation. Due to the short half-life of this substance, the discontinuation of adenosine infusion promptly eliminates the potential adverse effects [21].

In contrast, regadenoson is an A2a selective agonist, with minimal stimulation of A2b and A3 receptors. Like adenosine, it has a rapid onset of action; however, it is distinguished by a longer half-life. It presents a relatively safe profile, making it suitable for patients with chronic obstructive pulmonary disease. Nevertheless, despite the fact that regadenoson demonstrates high selectivity, its application might be linked to arrhythmia and the need for aminophylline administration [22].

It is important to note that both adenosine and regadenoson induce an increase in heart rate by 10–20 bpm, which might have an impact on image quality [18].

## 5. Examination Protocol

For a comprehensive analysis of myocardial perfusion, two distinct data sets are required: one obtained during rest, and the other acquired under pharmacologically induced stress (hyperemia). CT-MPI can be performed as a part of a CT protocol in combination with coronary CTA.

**Figure 1:**
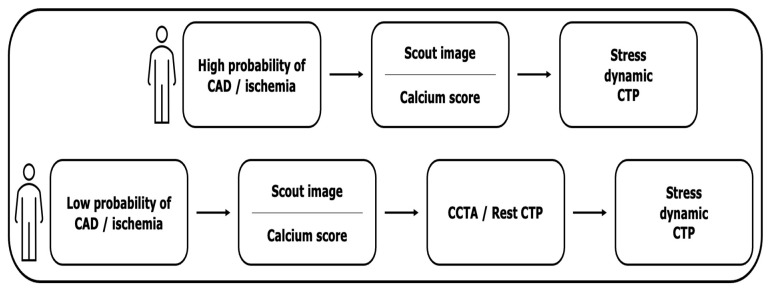
Dynamic CT-MPI image acquisition protocol.

Two distinct protocols are utilized, namely rest-stress and stress-rest. The optimal sequence of scans is still a matter of debate, and the diagnostic approach should be tailored to the individual risk profile [14,15]. Figure 1 depicts the acquisition protocols.

It is preferred to initiate the procedure with the stress CT-MPI in patients with a high probability of CAD or ischemia as well as a history of coronary revascularization (by-pass, stents), thus eliminating the contrast contamination as well as suppressing the effect of beta-blockers and nitroglycerin [23].

On the other hand, given that CCTA rules out significant coronary stenosis in the majority of patients with suspected CAD, the rest-stress CTP protocol is recommended for patients with a low-to-intermediate pretest probability of CAD to avoid stress examination and unnecessary radiation exposure in the absence of stenosis [14,15].

For the attainment of optimal contrast opacification and myocardial peak enhancement, it is recommended to administer contrast at a flow rate of 3–5 mL/s [24]. This flow rate ensures adequate and rapid delivery of contrast to the myocardium during the critical first pass phase, resulting in enhanced opacification of the myocardial vasculature and facilitating accurate evaluation of myocardial perfusion while detecting potential abnormalities [23,24]. The volume of iodinated contrast administered during CT-MPI is subject to variability depending on the specific protocol and clinical context but is typically in the range of 50–70 mL [15].

It is recommended to acquire images during a breath-hold period of 20 to 30 s in order to minimize respiratory motion artifacts, to achieve optimal contrast opacification, and to ensure image acquisition coincides with the peak arterial enhancement of the contrast agent in the myocardium [9].

Image acquisition in dynamic CT perfusion involves the use of prospective electrocardiogram (ECG) gating, which can be performed during either the systolic or diastolic phase. The optimal cardiac phase for image acquisition remains a topic of ongoing debate, with previous studies predominantly utilizing the diastolic phase. This preference is attributed to the fact that the myocardial perfusion of the left ventricle is more prominent in diastole, with the peak values being reached at the end of this phase. This phenomenon is related to the occurrence of coronary blood flow impedance during systole when the myocardium contraction induces reduced blood flow in the coronary vessels [14,18].

Nonetheless, there are advantages to acquiring images during systole, including the shorter basal-apical length enabling comprehensive heart coverage and the constant duration of systole, independent of heart rate. Therefore, while the diastolic phase is favored for myocardial perfusion evaluation, the systolic phase remains relevant in certain clinical scenarios, especially when comprehensive heart coverage is required. By implementing accelerated image acquisition protocols, such as high temporal resolution scanners, bolus tracking, and prospective ECG-gating, in addition to employing advanced motion-correction techniques, it is possible to mitigate the deleterious effects of motion artifacts during systole. These strategies collectively contribute to enhanced image quality and preservation of diagnostic accuracy [14,18].

## 6. Technical Aspects of Image Acquisition

Dynamic CT-MPI is based on the acquisition of multiple imaging datasets representing the variations in myocardial attenuation during the first pass of an injected contrast.

The information regarding the contrast passage, including the phases of inflow, peak concentration, and outflow is recorded.

The multi-phasic dataset is further utilized to generate time-attenuation curves for each voxel of the left ventricle and the reference arterial input function [9,15].

The acquisition of high-quality dynamic CT perfusion images is subject to several technical prerequisites. Temporal resolution is critical for optimal scanning during stress imaging, and sufficient *z*-axis coverage, determined by the width of the CT detector, is required to cover the LV myocardium.

Given these considerations, dynamic CT-MPI is only feasible with dedicated scanners that meet these specific criteria.

Currently, it is performed with dual-source CT (DSCT) scanners and wide multidetector CT (MDCT) systems [25].

DSCT systems, equipped with two X-ray tubes and corresponding detectors, provide high temporal resolution (75 or 66 ms) without the need for faster gantry rotation. However, while DSCT scanners offer a temporal advantage, it is important to note that the introduction of shuttle mode might be required to achieve sufficient *z*-axis coverage as the DSCT cannot cover the entire heart in a single rotation. The shuttle mode, which merges two acquisitions into a single image, can achieve a *z*-axis coverage of 7.3 cm (second generation) or 10.5 cm (third generation) [25]. On the other hand, the shuttle mode is inherently related to the motion artifact, which may influence the quantification of MBF.

In contrast, higher gantry rotation speed is essential to improve temporal resolution in MDCT systems with a wide detector array of 256 or 320 rows. This setup provides full heart coverage (*z*-axis coverage of 16 cm) while the table remains stationary. The elimination of the shuttle mode reduces the time required for image acquisition while increasing the number of images obtained, allowing for a more detailed analysis of contrast dynamics. However, each additional image acquired is associated with a corresponding increase in the radiation dose [26,27].

## 7. Image Analysis

Dynamic CT perfusion serves as a tool for assessing myocardial perfusion using either a semi-quantitative or fully quantitative approach.

The attenuation values of each pixel in the myocardium are evaluated over time to generate TACs [28]. The TACs are further utilized to derive indirect perfusion parameters such as peak enhancement, time to peak (TTP), tissue transit time (TTT), attenuation upslope, and area under the curve (AUC). In regions of ischemia or infarction, which indicate impaired blood flow, peak enhancement and AUC values are decreased [18].

As there is a direct correlation between CT attenuation and iodine concentration, the upslope of the TAC in the myocardium exhibits a strong linear correlation with the myocardial blood flow (MBF) [29].

There are several methods for analyzing TACs and quantifying blood flow within the myocardial tissue. Two commonly used methods for dynamic CT flow calculation are the upslope method and the model-based deconvolution technique.

The upslope method has notable advantages in terms of robustness and feasibility [30]. However, it is limited by its semi-quantitative nature, providing relative measurements, and lacking absolute blood flow values. Furthermore, the sensitivity of this method is influenced by various factors such as contrast injection techniques and scan timing, which can introduce errors and compromise the reliability of the results. Consequently, its ability to detect subtle perfusion changes in ischemic or infarcted regions may be diminished [31].

To address these limitations, more complex deconvolution techniques have been developed to permit a fully quantitative assessment of myocardial perfusion. TACs are processed mathematically and coupled with the arterial input function (AIF) using a hybrid deconvolution model to calculate the perfusion parameters [32].

The deconvolution technique, based on the two-compartment model, offers the advantage of absolute quantification by accounting for blood flow into the myocardium, myocardial blood flow itself, and the rate of contrast extraction into the interstitial space. The volumetric image dataset is subsequently processed [10,33]. However, it is important to note that challenges related to contrast administration and accurate determination of the arterial input function (AIF) need to be addressed to ensure accurate quantification of perfusion parameters.

There is a limited amount of data on the variability of AIF site selection and its subsequent impact on MBF quantification in dynamic CT-MPI.

Differences in PET-MBF values have been demonstrated for different AIF locations. These variations have been attributed to the partial volume effect, the size of the ROI used for AIF, motion, respiratory artifacts, and adjacency to vascular structures [34].

In addition, the heterogeneity of perfusion within the myocardium and the occurrence of bolus dispersion and transit delay can also affect the accuracy of the shape and intensity of the AIF curve [35].

While further research is warranted to explore the role of variable AI sites in dynamic CT-MPI, it is reasonable to infer that the use of fixed AIF could potentially lead to inaccurate estimation of MBF. Therefore, the choice of AIF site should be tailored to each patient [34,35].

**Figure 2:**
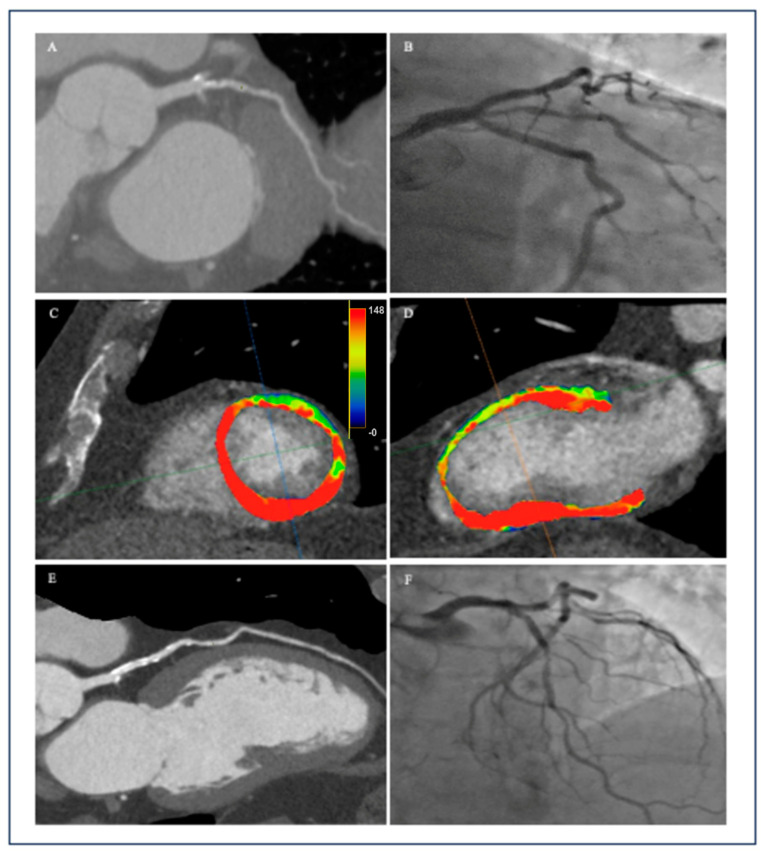
A 68-year-old male with atypical angina and the following cardiovascular risk factors; hypertension, diabetes type 2, hyperlipidemia, nicotine dependence, obesity (BMI 33 kg/m^2^). The patient underwent CT coronary angiography, dynamic CT myocardial perfusion, and invasive coronary angiography. Coronary CT angiography revealed: 65% luminal narrowing [CAD-RADS: 3 in the ramus intermedius artery (**A**); 65% luminal narrowing [CAD-RADS: 3/N] in the left anterior descending artery (**E**). Invasive coronary angiography revealed: 75% stenosis in the proximal ramus intermedius artery (**B**); 30% stenosis in proximal LAD, 70% stenosis in mid-LAD, and severe stenosis in diagonal branches (**F**). Regadenoson-stress myocardial CT perfusion with dynamic acquisition: ((**C**)—short axis and (**D**)—2-chamber view) revealed perfusion defect with decreased MBF in the mid anterior, mid-anterolateral, and apical anterior segment. Scan protocol included: 400 mg of regadenoson and 45 mL of nonionic iodinated contrast. FFR measurements performed during ICA-FFR value of 0.75 indicating significant changes in LAD and FFR value of 0.78 indicating significant changes in the intermediate branch.

Color-coded volumetric perfusion maps, illustrated in Figure 2, provide a visual representation of the quantitative analysis results. While the primary approach to dynamic CTP image analysis involves visual assessment of perfusion defects, incorporating quantitative analysis of perfusion parameters offers valuable insights. Specifically, a reduced value in myocardial blood flow (MBF) signifies a myocardial perfusion defect, potentially indicating ischemia. It is worth noting that a definitive cut-off value for hyperemic MBF in diagnosing hemodynamically significant stenosis has yet to be established.

## 8. Image Quality

Beam hardening artifacts, a common problem in CT imaging, result from the selective absorption of low-energy photons as the X-ray wave passes through objects of varying densities. This phenomenon leads to the appearance of a hypoenhanced area that can be misinterpreted as a false-positive perfusion abnormality [15]. In the context of CT-MPI, a notable consideration lies in the high iodine concentration in the ventricular cavity and descending aorta. Other contributing factors include metallic implants, dense calcifications within blood vessels, bony structures, implanted medical devices, and scattering effects. These artifacts manifest as dark bands on contrast-enhanced images, predominantly affecting the inferior and anterior walls of the left ventricle [15]. Iterative reconstruction (IR) techniques have been employed to provide enhanced image quality while concurrently reducing radiation dose [36]. This is particularly important in the context of the high radiation exposure associated with dynamic CT-MPI and the image noise inherent in low-dose protocols.

Notably, the application of Statistical Iterative Reconstruction (SIR) is noteworthy for its ability to eliminate streaking artifacts found in dynamic CT-MPI datasets acquired using low tube currents [37]. Furthermore, the contrast-to-noise ratio (CNR) and the signal-to noise ratio (SNR) are also improved with the implementation of IR [38].

Simultaneously, the Adaptive Statistical Iterative Reconstruction (ASIR) effectively minimizes noise-related errors in low-dose acquisition protocols and facilitates quantitative myocardial perfusion measurements. Therefore, it has been suggested that dynamic CT-MPI utilized with the ASIR represents a promising alternative to MRI and SPECT for the assessment of myocardial ischemia [39].

Although iterative reconstruction (IR) techniques are effective in reducing beam hardening artifacts and improving spatial denoising, they may not inherently improve temporal smoothness. Interestingly, the recent incorporation of four-dimensional noise reduction filtering utilizing a similarity algorithm (4D-SF) in conjunction with IR has been shown to improve image quality and maintain accurate CT-MBF quantification in dynamic CTP-MPI [40].

The resulting images have a more realistic texture and sharper contours than images taken with IR alone [40,41].

Conversely, Deep Learning Image Reconstruction (DLIR) employs deep convolutional neural networks to overcome the inherent modeling limitations of IR methods. DLIR has demonstrated remarkable performance in suppressing image noise, preserving structural details, and detecting lesions, surpassing the capabilities of conventional FBP [42].

It has been shown that the implementation of a deformable deep learning-based image registration method in dynamic CT-MPI, which incorporates a recursive cascade network in addition to a ventricular segmentation module and a novel loss function, effectively achieves the registration of dynamic cardiac perfusion sequences by mitigating local tissue displacements. Given the preservation of image quality and the short processing time, it has the potential to be integrated into routine quantitative CT myocardial perfusion measurements [43].

## 9. Diagnostic Accuracy of Dynamic CTP-MPI in the Diagnosis of Myocardial Ischemia

Dynamic CT-MPI exhibits strong concordance with established reference methods, including MRI, SPECT, and PET. It demonstrates a sensitivity of 0.93 (0.82–0.98, 95% CI interval) and a specificity of 0.82 (0.70–0.91, 95% CI interval) in detecting myocardial ischemia [44].

This imaging modality has been extensively evaluated for its efficacy with several clinical trials using various stressor agents and standard reference methods demonstrating its feasibility and validity [45,46,47,48,49,50,51]. Table 1 provides a comprehensive overview of studies related to dynamic CT perfusion imaging.

Quantitative assessment of myocardial perfusion aids in the characterization of myocardial ischemia. The analysis of the perfusion defects holds the potential to differentiate between ischemic and infarcted myocardium [52]. Qualitative and semi-quantitative myocardial perfusion parameters correlate strongly with MRI in differentiating ischemic from non-ischemic myocardium, with sensitivity, specificity, positive and negative predictive values of 86.1%, 98.2%, 93.9%, and 95.7%, respectively [53].

Given that dynamic CTP provides information on the hemodynamic significance of coronary artery stenosis, it can serve as an effective tool in the diagnosis of CAD.

Synergistic use of CCTA and CTP-MPI provides higher diagnostic accuracy compared to CCTA alone in both per segment (96 versus 68%, *p* = 0.002) and per vessel (95% versus 75%, *p* = 0.012) analyses [54]. The combined approach of CTP-MPI with CCTA yields a significantly enhanced AUC of 0.878 compared to an AUC of 0.826 (*p* < 0.05) for CCTA alone in identifying flow obstructing stenoses [55].

CCTA and CTP, when concordant, accurately confirm or exclude ischemia [56]. The inclusion of indexed MBF increases the positive predictive value for the detection of hemodynamically relevant coronary stenoses from 49% (for CCTA alone) to 78% [57].

It is crucial to acknowledge that the diagnostic specificity of CCTA may be constrained by various factors, including a high calcium score, the presence of stents or bypass, and a notable incidence of intermediate stenosis of unknown hemodynamic significance [58,59]. The visual assessment of coronary stents can be challenging due to the occurrence of blooming and beam hardening artifacts induced by metallic stent struts and the extensive atherosclerotic burden of unstented segments [58]. These artifacts substantially compromise image quality, inadvertently leading to an overestimation of arterial stenosis and the generation of false positive results.

However, it is crucial to note that these confounding factors exert minimal or no impact on myocardial perfusion, underscoring the role of dynamic CTP as an essential adjunct to diagnostic evaluation.

In patients with severely calcified lesions (CAC score ≥ 400), the combined CCTA-CTP-MPI approach demonstrates superior diagnostic accuracy when compared to the use of CCTA or CTP-MPI alone [60].

Information on the functional significance of the coronary lesion provided by dynamic CTP can be used to guide treatment decisions and the need for revascularization [56]. Notably, the CTP-MPI-CCTA-guided approach demonstrates a significantly lower rate of ICA as compared to the CCTA-guided approach (48.3% versus 30.8%, *p* = 0.006), with no significant differences observed in the incidence of major adverse cardiac events (MACE) between the two groups [61]. In addition, the integration of indexed MBF values has been shown to have a significant impact on the diagnostic process, resulting in the reclassification of 43% of coronary lesions and subsequent management changes [57].

Furthermore, myocardial perfusion CT emerges as a strong predictor of MACE, surpassing the value of clinical risk factors. Patients with at least one perfusion defect have a significantly higher likelihood of MACE, with a hazard ratio of 2.50 (95% CI of 1.34–4.65, *p* = 0.0040) [62].

**Table 1:** Summary of Dynamic CT Perfusion Imagining Studies.

Authors and the Year	Study Population (n = Number of Patients)	Scanner Type	Reference Modality/ Comparator	Abnormal Hyperaemic MBF [mL/100 mL/min]	Sensitivity [%]	Specificity [%]
Kim et al., 2014 [45]	33	128-slice Dual Source (Siemens)	MRI	62	81 (per myocardial segment) 82 (per-territory)	94 (per myocardial segment) 84 (per-territory)
Ho et al., 2010 [46]	35	128-slice Dual-source (Siemens)	SPECT	57–65	83	78
Rossi et al., 2017 [51]	115	Second-generation dual-source (Siemens)	ICA and FFR	92 (74–109)	75	88
Bamberg et al., 2014 [52]	31	Dual-source (Siemens)	MRI	88 (any perfusion defect) In high-quality examinations: 72 for ischemia and 73 for infarction.	78	75
Bastarrikka et al., 2010 [53]	10	Second-generation dual-source (Siemens)	MRI	96	86	98
Kono et al., 2014 [63]	42	128-slice dual-source (Siemens)	FFR	76	98	70
Pontone et al., 2019 [55]	85	Third generation (GE Revolution)	ICA and FFR	96	73	86
Baxa et al., 2015 [54]	54	Second-generation dual-source (Siemens)	CCTA + ICA		97	95
Lubbers et al., 2018 [64]	268	Second and Third generation dual-source (Siemens)	Functional testing (95% exercise ECG)		-	-
Meinel et al., 2017 [62]	242	Second-generation dual-source (Siemens)	CCTA	-	-	-
Kurata et al., 2013 [50]	9	256 MDCT (Philips)	ICA	110–184	75	87
Nous et al., 2022 [47]	114	Third-generation dual-source (Siemens)	ICA + FFR	142 (absolute MBF) 0.82 (relative MBF)	84 (per patient) 89 (per vessel)	89 (per patient) 79 (per vessel)
Alessio et al., 2019 [65]	34	256-slice system (GE Revolution)	PET	-	75	83
Wang et al., 2012 [66]	30	128-MDCT dual-source (Siemens)	SPECT	90	90	81
Kikuchi et al., 2014 [49]	32	320-row MDCT (Toshiba)	PET		86	92

## 10. Limitations

Dynamic CT perfusion is an evolving imaging modality. Its use is currently limited to the scientific community and a few research centers since it can only be performed on advanced CT scanners. As a result, most of the available research data come from studies using shuttle-mode DSCT scanners, which are potentially prone to motion artifacts.

The primary constraint of this technology, however, is its heterogeneity. There are no standardized protocols for scan acquisition and image analysis. Quantification of absolute myocardial blood flow (MBF) values tends to be underestimated due to a combination of technical factors and patient-specific characteristics. This lack of uniformity hinders effective comparison of absolute MBF values and may result in disease thresholds lacking generalizability.

Given that dynamic CT perfusion requires the acquisition of multiple consecutive phases, it is associated with a notable radiation exposure. Various strategies have been employed to reduce radiation exposure, such as adjusting the tube voltage or reducing the temporal sampling rate. However, these approaches could potentially introduce distortions in the accurate estimation of MBF, consequently affecting the accuracy of the results [67].

Furthermore, it is crucial to emphasize the paucity of studies addressing the consistency of CTP data over time. Further research is imperative to investigate the reproducibility and validity of CTP measurements, to ensure their reliability for clinical use.

## 11. Conclusions

Clinical research suggests that the integration of CTP-MPI with CCTA has the potential to become a one-stop-shop method for the accurate identification of obstructive CAD.

In the regions of concordance between CCTA and CTP, the combined approach of CCTA-CTP demonstrates high accuracy in the detection and exclusion of myocardial ischemia and thus can act as an effective gatekeeper to invasive procedures.

In addition, this combined approach allows precise analysis in scenarios where the value of anatomical assessment is limited, such as previous stent implantation, severely calcified lesions, and high coronary plaque burden, making CTP an excellent approach to overcome the limitations of CCTA.

## 12. Future Perspectives

However, it should be noted, that dynamic CTP-MPI has not yet been incorporated into any clinical guidelines. In contrast, an alternative approach to assess the hemodynamic significance of coronary lesions, namely CT-FFR, has already earned a place in the recent ACC/AHA guidelines [68]. This discrepancy underscores an important point: while functional assessment is playing an increasingly important role in the diagnosis of CAD, several prerequisites must be me for the dynamic CT-MPI to become an accepted technique. These include the establishment of a standardized acquisition protocol and the validation of perfusion parameters. In addition, the development of innovative reconstruction techniques coupled with AI support is necessary to improve image quality and reduce radiation dose, which remains a concern for this modality.

## Data Availability

Not applicable.
